# An assessment of the pH changes and metal ions released into artificial saliva by fake orthodontic braces

**DOI:** 10.1186/s12903-023-03339-7

**Published:** 2023-09-16

**Authors:** Riyam Haleem, Noor Ayuni Ahmad Shafiai, Siti Noor Fazliah Mohd Noor

**Affiliations:** 1 Department of Dentistry, Al-Hadi University College, Baghdad, 10011 Iraq; 2 Department of Dental Science, Advanced Medical and Dental Institute (AMDI), Universiti Sains Malaysia, Kepala Batas, Pulau Pinang 13200 Malaysia

**Keywords:** Biomaterial, Counterfeit, Ions released, Orthodontic braces

## Abstract

**Background:**

This present study assesses changes in the pH as well as the metal ions that fake braces release into artificial saliva (AS) using a pH meter and inductively coupled plasma atomic emission spectroscopy (ICP-AES), respectively.

**Methods:**

Three sets of fake archwires (AWs) and brackets (Bs) as well as a set of controls were immersed in AS and placed in an incubator shaker at 50 rpm and 37°C. At Days 0, 1, 7, 14, 21, and 28, the pH of the AS medium was measured and 3.0 ml of AS was collected and stored at -20°C for elemental analysis.

**Results:**

Significant changes in pH were observed on Days 0, 1, 7, 14, 21, and 28 in the AS of the AW group. However, these changes were only observed in the B group on Days 0 and 7. The fake samples released a large quantity of sodium (Na), potassium (K), and calcium (Ca) ions, at concentrations exceeding 100 mg/L, post-28 days of immersion. The control and fake braces samples released other ions; such as lithium (Li), magnesium (Mg), barium (Ba), chromium (Cr), copper (Cu), lead (Pb), and aluminium (Al); at concentrations that did not exceed 10 mg/L.

**Conclusions:**

The pH of the AS of all the samples increased post-incubation. Only 10 ions; namely, Na, Li, K, Mg, Ca, Ba, Cr, Cu, Pb, and Al; were detected in the AS.

## Background

There is a growing fashion trend of donning fake orthodontic braces in Southeast Asia. Fake is generally defined as not true, not real, not genuine, or artificial [1]. In terms of dentistry, fake braces are ‘orthodontic braces’ that are often sold online or are available from uncertified sellers at much cheaper prices. They are often self-installed or installed by unqualified individuals. Furthermore, proper dental examinations or investigations; such as radiographs or model studies; are not conducted before the fake braces are affixed [2]. The British Dental Association (BDA) is concerned by this service as any type of orthodontic procedure first warrants an oral examination to determine the oral condition of the patient and if the patients has any existing dental disease that should be treated prior to orthodontic procedure followed by a treatment plan [3]. Without careful professional dental supervision, an orthodontic procedure can lead to caries and gingival bleeding [4], thereby, worsening the oral health of the patient. Reports of the severe negative effects of fake braces have emerged. For instance, two Thai teenagers succumbed to infections after wearing fake braces [5]. In fake braces, the archwires (AWs) range from plain ligature wires to twisted multi-ligature wires, figure eights, and nickel titanium (NiTi) wires supported by ring positioning or elastomeric chains (E chains) of different colours and patterns. The brackets (Bs) are made of ceramics or metals and without or with hooks and vary in size.

Fake braces are popular among teenagers as they are a cheap alternative to wearing orthodontic devices. Furthermore, they are removable as they are often loosely affixed by hooks, self-glued, or fixed using self-setting glue. Although 60% of teenagers are aware of do-it-yourself (DIY) braces, almost half of them do not know the difference between actual and fake braces [6]. A recent study of 10 subjects who wore fake braces revealed that fake braces are popular as they are easy to access, convenient, and reasonably priced as they are provided by bogus dentists. Although some did not observe any changes after wearing fake braces, most felt that the condition of their teeth had worsened [7].

The Internet and social media are the main sources of information on fake braces [3, 6]. Patients are also known to show an Internet image or a picture of their friend's fake braces and demand similar Bs, attachments, and plastic chain patterns [8]. Most fake braces suppliers carry a wide range of E chains and modules to satisfy their clients’ colour and pattern demands. These comprise every colour and theme possible; from flowers to power O chain braces and Hello Kitty to Mickey Mouse themed braces [8]. These fake braces are, typically, applied primarily and not completely to the maxillary arch. Active and passive fake braces are the two most common types of fake braces available. Uncertified individuals use etching and bonding techniques to mount fake braces to the teeth. While active fake braces are bonded to the teeth, passive fake braces are not attached to the teeth and are easily worn and removed. Attention-grabbing keywords such as easily worn, removable, does not move the teeth, and accessories are often used to advertise passive fake braces or ‘click braces’ online. These types of braces are equipped with a clasp that temporarily anchors the braces to the molars, with customers expected to wear these braces only for a limited period of time [9]. The composition of these fake braces is unknown as they are never stated online. However, metal ion leaching is a concern as excessive levels of these ions can result in many health issues or toxicity [10].

An extant study assessed the leaching of metal ions from these fake braces into simulated bodily fluid [11]. This present study aimed to explore the metal ion leaching of fake braces as well as the changes that they cause to the pH of artificial saliva (AS). As fake braces are placed in the oral cavity, AS may provide a more accurate representation of the oral condition of patients wearing fake braces. An inductively coupled plasma optical emission spectroscopy (ICP-OES) was conducted to analyse the ions that the fake braces released into the AS.

## Methods

### Sample size determination

The release of nickel (Ni) ions is the biggest concern as it can cause allergic reactions. Therefore, assuming that stainless steel orthodontic AWs contain 8% Ni [12], the sample size was calculated using a single proportion formula to detect differences between the amount of Ni ions released by the fake braces' samples and the control samples. At 80% power with 5% level of significance, a total of three samples per groups was sufficient.

### Braces sample preparation

The keywords ‘fake braces’, ‘click braces’, and ‘click power chain’ were searched on an online shopping platform (www.shopee.com.my) to identify and purchase the three fake braces' samples. The search results showed the names of different suppliers and items with the highest sales, that were most likely the most purchased as they were either cheap or in high demand or popular among buyers [11].

The orthodontic devices were disassembled for the pH evaluation and ICP-OES analysis. Each type of fake braces was disassembled into four brackets (B) and four archwires (AW) that had each been cut into 10 mm lengths. The control sample comprised a 0.018-inch 3 M™ Unitek™ stainless steel archwire and an orthodontic metal bracket (mini MBT 022, Hook 345). Each sample was weighed prior to soaking in the AS (Table 1).

**Table 1:** Weights of samples in milligrams (mg)

	Control Mean (SD)	Type 1 Mean (SD)	Type 2 Mean (SD)	Type 3 Mean (SD)	*P-*value^a^
Bracket	67.8 (0.59)	61.3 (1.06)	53.0 (1.03)	52.8 (0.95)	0.09
Archwire	13.4 (0.09)	13.5 (0.24)	23.4 (0.33)	6.8 (0.08)	0.82

^a^One-sample t test

### Artificial saliva (AS) medium preparation

The AS medium was prepared according to Amal et al. [13] at pH 7.4. Table 2 shows its composition. The amount of AS required for each sample was determined based on the weight of the sample. The AS concentration for the AW was 0.5 mg/ml and that of the B was 2 mg/ml. The AWs and Bs were separately immersed in the AS and placed in an IKA® KS 4000 incubator shaker at 50 rpm and 37°C to mimic the salivary flow and temperature of the human body.

**Table 2:** Weight and volume of reagents for 1000 ml of AS

Component	Amount
NaCl	400 mg
KCl	400 mg
CaCl_2_.2H_2_O	795 mg
NaH_2_PO_4_.H_2_O	1.6 mg
Na_2_S.9H_2_O	5 mg
Urea	1000 mg
KSCN	300 mg

### pH analysis

On Days 0, 7, 14, 21, and 28, a Metrohm™ pH meter was used to measure the pH of the AS containing the immersed samples. Prior to use, the pH meter was calibrated for pH 4 and 7, according to the standard NIST pH buffering solution.

### Elemental analysis

Following the pH evaluations, on Days 0 (pre-sample immersion baseline), 7, 14, and 28, 3.0 ml of the AS was also collected from every sample and stored at -20°C in a freezer for the ICP-OES analysis. On the day of the analysis, the samples were thawed to room temperature, filtered using a 0.22-μm syringe filter, and diluted by a factor of 10 in deionised water. A PerkinElmer® Optima™ 8000 ICP-OES was used to conduct the elemental analysis of the control and fake braces samples. The standard calibration curve of each ion was obtained by preparing a standard solution of Sigma-Aldrich® Multielement Standard Solution 6 for ICP, which contains 23 elements; such as lithium (Li), potassium (K), sodium (Na), magnesium (Mg), barium (Ba), calcium (Ca), chromium (Cr), copper (Cu), lead (Pb), and aluminium (Al) ions; between 0 to 40 ppm, with quality control checks at 7.5 and 12.5 ppm, according to the manufacturer’s instructions (Table 3). This was to ensure that the concentrations of metallic ions released were within the range of the instrument. Each sample was measured in triplicate.

**Table 3:** List of elements with their absorption wavelengths used in the ICP-OES

Element	Wavelength (nm)
Calcium (Ca)	422.673
Cobalt (Co)	238.892
Cadmium (Cd)	228.802
Iron (Fe)	259.939
Nickel (Ni)	231.604
Lead (Pb)	283.306
Zinc (Zn)	213.857
Chromium (Cr)	357.869
Magnesium (Mg)	285.213
Copper (Cu)	324.752
Vanadium (V)	311.071
Titanium (Ti)	368.519
Antimony (Sb)	217.582
Molybdenum (Mo)	281.616
Sodium (Na)	589.592
Lithium (Li)	670.784
Aluminium (Al)	309.271
Boron (B)	182.578
Barium (Ba)	455.403
Potassium (K)	766.490
Manganese (Mn)	257.610
Phosphorus (P)	214.914
Silicon (Si)	251.611

### Statistical analyses

The data was compiled in a Microsoft® Excel sheet and analysed with IBM® SPSS® Statistics 26, at 5% significance. Descriptive analyses, Bonferroni post-hoc tests, and one-way analysis of variance (ANOVA) were used to determine differences between the metal ions that the fake and control braces samples released in the AS.

## Results

### Changes in the pH of the artificial saliva (AS)

**Fig. 1:**
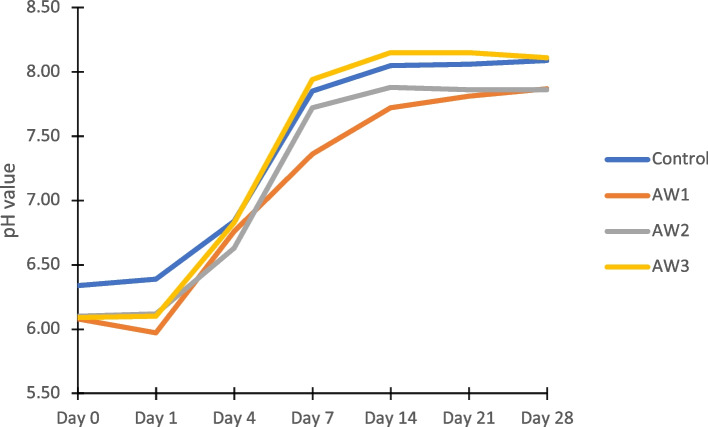
pH behaviour of control and fake AW during incubation in AS

Figure 1 depicts changes in the pH of the AS of the control and fake AW samples during incubation. The pH of all the samples increased post-incubation. The pH of the control AW rose from 6.34 on Day 0 to 8.09 on Day 28. Unlike the pH of the control AW and the other fake AWs, the pH of AW1 rose the least; specifically, 7.36 on Day 7 to 7.87 on Day 28. Furthermore, the pH of AW3 rose from 6.09 on Day 0 to 8.15 on Days 14 and 21 before it decreased to 8.11 on Day 28. Significant differences in the pH of each group were observed on Days 0, 1, 7, 14, 21, and 28 (Table 4).

**Table 4:** Mean pH values of control and fake archwires during incubation in AS

	**pH values**				***F* Statistic (*df*)**	***P* value**^**a**^
	**Mean (SD)**					
	Control (*N* = 3)	AW1 (*N* = 3)	AW2 (*N* = 3)	AW3 (*N* = 3)		
Day 0	6.34 (0.09)	6.08 (0.10)	6.10 (0.03)	6.09 (0.04)	5.97 (5, 12)	0.005*
Day 1	6.39 (0.06)	5.97 (0.10)	6.12 (0.01)	6.10 (0.02)	16.74 (5, 12)	0.000*
Day 4	6.84 (0.17)	6.76 (0.09)	6.63 (0.08)	6.83 (0.17)	0.79 (5, 12)	0.577
Day 7	7.85 (0.25)	7.36 (0.19)	7.72 (0.16)	7.94 (0.28)	4.89 (5, 12)	0.011*
Day 14	8.05 (0.03)	7.72 (0.11)	7.88 (0.04)	8.15 (0.02)	26.47 (5, 12)	0.000*
Day 21	8.06 (0.05)	7.81 (0.14)	7.86 (0.02)	8.15 (0.02)	11.64 (5, 12)	0.000*
Day 28	8.09 (0.02)	7.87 (0.11)	7.86 (0.05)	8.11 (0.20)	4.13 (5, 12)	0.021*

^*^The mean difference is significant at the 0.05 level

^a^One-way ANOVA

**Fig. 2:**
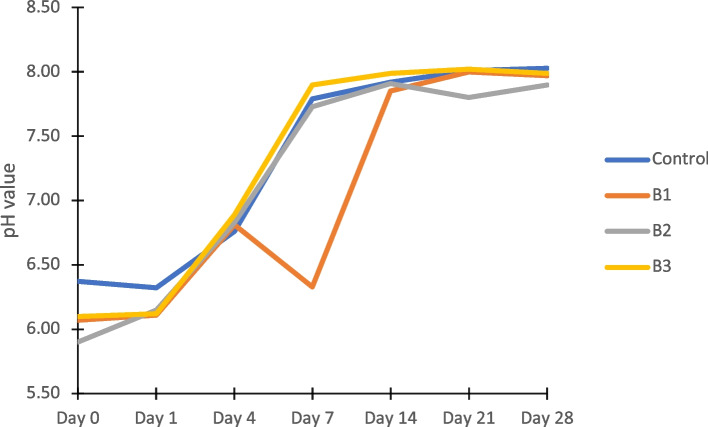
pH behaviour of control and fake brackets during incubation in AS

Figure 2 depicts changes in the pH of the AS of the control and fake B samples over 28 days. The pH of the control B increased from 6.37 on Day 0 to 8.03 on Day 28. The pH of B1 was lower than that of the control B, B2, and B3 samples on Day 7. However, it rose to 7.85 on Day 14 and behaved similarly as the control B and the other fake Bs until Day 28. Therefore, the pH of the groups only differed significantly on Days 0 and 7 (Table 5).

**Table 5:** Mean pH values of control and fake brackets during incubation in AS

	**pH values**				***F* Statistic (*df*)**	***P* value**^**a**^
	**Mean (SD)**					
	Control (*N* = 3)	B1 (*N* = 3)	B2 (*N* = 3)	B3 (*N* = 3)		
Day 0	6.37 (0.02)	6.07 (0.23)	5.90 (0.16)	6.10 (0.08)	6.06 (5, 12)	0.005*
Day 1	6.32 (0.08)	6.11 (0.26)	6.15 (0.06)	6.12 (0.05)	0.000 (5, 12)	1.000
Day 4	6.76 (0.15)	6.81 (0.22)	6.83 (0.07)	6.89 (0.10)	0.75 (5, 12)	0.605
Day 7	7.79 (0.21)	6.33 (0.11)	7.73 (0.12)	7.90 (0.12)	8.77 (5, 12)	0.001*
Day 14	7.92 (0.10)	7.85 (0.32)	7.91 (0.03)	7.99 (0.04)	1.41 (5, 12)	0.288
Day 21	8.01 (0.02)	8.00 (0.15)	7.80 (0.12)	8.02 (0.04)	2.84 (5, 12)	0.064
Day 28	8.03 (0.03)	7.97 (0.13)	7.90 (0.01)	7.99 (0.06)	1.75 (5, 12)	0.197

^*^The mean difference is significant at the 0.05 level

^a^One-way ANOVA

### Ions released in the artificial saliva (AS) by the Fake Braces 

The fake and control Bs released Na, Li, K, Mg, Ca, Ba, Cr, Cu, Pb, and Al ions. The largest quantity of ions released by the fake Bs comprised Na, K, and Ca ions, at concentrations exceeding 100 mg/L after 28 days of immersion. Moreover, the quantity of Na ions released by the control and fake Bs significantly increased on Day 7 then plateaued until Day 14 before significantly increasing on Day 28. Tables 6, 7, and 8 depict the mean concentrations and significant differences in the quantity of Ca, Na, and K released, respectively. The concentrations of other ions; such as Li, Mg, Ba, Cr, Cu, Pb, and Al; that the control and fake Bs released into the AS were all less than 10 mg/L.

**Table 6:** Ca ions released (mg/L) during 28 days of immersion presented as mean (SD)

	Control	Type 1	Type 2	Type 3	*F* statistic (df)	*P* value
Brackets (B)						
Day 7	191.85 (1.60)	186.55 (2.08)	177.45* (2.62)	174.05* (1.28)	50.34 (3,8)	0.000
Day 14	188.60 (1.80)	147.17* (0.72)	188.75 (1.00)	170.15* (2.84)	364.95 (3,8)	0.000
Day 28	203.75 (2.67)	205.85* (1.0)	194.35* (2.35)	173.05* (1.13)	7107.79 (3,8)	0.000
Archwires (AW)						
Day 7	202.95 (0.79)	194.35* (0.75)	192.3* (0.26)	164.4* (1.28)	1152.27 (3,8)	0.000
Day 14	196.45* (0.53)	202.15 (2.10)	196.7* (2.61)	171.65 (0.62)	188.02 (3,8)	0.000
Day 28	199.25 (2.72)	192.75 (1.88)	195.7 (3.16)	160.8* (0.79)	175.28 (3,8)	0.000

^*^Statistically significant difference between control and fake samples by using Bonferroni post hoc test, *P* < 0.05

^a^ One−way ANOVA

**Table 7:** Na ions released (mg/L) during 28 days of immersion presented as mean (SD)

	Control	Type 1	Type 2	Type 3	*F* statistic (df)	*P* value
Brackets (B)						
Day 7	142.71 (1.29)	119.10* (0.34)	100.99* (0.99)	117.09* (2.81)	334.38 (3,8)	0.000
Day 14	127.07 (2.05)	107.12* (1.60)	128.17 (1.08)	117.93* (1.05)	125.29 (3,8)	0.000
Day 28	137.84 (1.63)	138.45 (1.02)	123.47* (1.62)	117.24* (1.99)	173.88 (3,8)	0.000
Archwires (AW)						
Day 7	135.19 (0.93)	138.14 (1.94)	128.81* (0.64)	99.67* (1.40)	541.63 (3,8)	0.000
Day 14	135.48 (0.44)	136.93 (0.80)	130.87 (2.56)	112.27* (2.31)	119.01 (3,8)	0.000
Day 28	135.27 (0.95)	129.90* (1.17)	134.31 (1.93)	110.12* (1.34)	206.74 (3,8)	0.000

^*^Statistically significant difference between control and fake samples by using Bonferroni post hoc test, *P* < 0.05

^a^ One−way ANOVA

**Table 8:** K ions released (mg/L) during 28 days of immersion presented as mean (SD)

	Control	Type 1	Type 2	Type 3	*F* statistic (df)	*P* value
Brackets (B)						
Day 7	271.4 (2.65)	223.55* (1.48)	179.60* (1.54)	242.90* (1.21)	1366.42 (3,8)	0.000
Day 14	210.30 (1.67)	206.60 (0.23)	236.65* (1.28)	214.20 (2.93)	167.76 (3,8)	0.000
Day 28	269.35 (1.48)	275.50* (0.96)	254.75* (1.43)	251.00* (0.61)	295.76 (3,8)	0.000
Archwires (AW)						
Day 7	264.40 (2.19)	256.60* (2.42)	252.20* (0.88)	196.20* (2.89)	589.11 (3,8)	0.000
Day 14	268.40 (0.90)	270.35 (1.58)	259.90* (2.70)	239.35* (3.18)	116.38 (3,8)	0.000
Day 28	280.65 (1.20)	253.70* (4.13)	268.50* (2.86)	230.15* (0.98)	205.38 (3,8)	0.000

^*^Statistically significant difference between control and fake samples by using Bonferroni post hoc test, *P* < 0.05

^a^ One−way ANOVA

## Discussion

This present study used AS as the immersion medium as it works the same way as natural saliva to continuously lubricate and moisturise the mouth [14, 15]. As a fake orthodontic appliance will come into contact with the saliva in a mouth, any metal ions that leach will immediately be present in the saliva. As such, AS was used to mimic the natural condition of the human mouth [16]. Although the best test subjects would have been patients fitted with fake braces, it would have been unethical to recruit individuals and fit them with fake braces and it was difficult to locate patients who already wear fake braces as fake braces are illegal.

Interestingly, the pH of the AS of all the samples; the control, the fake Bs, and the AWs; rose from 5.90 to 8.11 when immersed in the AS. Peros et al. [17], similarly, reported an increase; from 7.18 to 7.30; in the pH of three separate stimulated saliva samples of 23 patients collected every six weeks following an AW sequence of 0.012-inch nickel-titanium (NiTi), 0.016-inch NiTi, and 0.016-inch by 0.022-inch NiTi. However, Arab et al. [18] reported a decrease; from 7.18 to 6.81; in the pH of the unstimulated saliva samples of 30 orthodontic patients wearing a fixed orthodontic appliance of 0.022 inch-slot NiTi wire and ‘Ameri-can’ brackets for 18 weeks. The differences in the pH, towards a more acidic or alkaline state, may have been due to the method of collecting the stimulated and unstimulated saliva samples, as unstimulated saliva may contain bacteria that is dominant in the oral ecosystem.

This present study used an in vitro set-up, where the pH and temperature were controlled with no interference from mastication and speech activities. Therefore, the observed changes in pH were entirely due to the orthodontic devices examined. The ions that the fake braces released into the AS may have increased the pH of the AS as it reacted with the heavy metal oxides [19]. An increase in the soluble metal hydroxide content of AS can produce an AS solution that is alkaline. According to Kuhta et al. [20], the oral environment facilitates the corrosion and biodegradation of dental materials as it experiences mechanical, chemical, thermal, enzymatic, and microbiological changes. Therefore, fake braces may increase pH changes in the oral cavity.

The finding of this present study, that fake braces increase the pH of AS, is significant for oral health as a few studies suggest that higher pH facilitates higher dental plaque mineralisation [21, 22]. Patel et al. [22] categorised patients as either gingivitis, periodontally healthy, and chronic periodontitis patients and found that the pH of the saliva samples of chronic periodontitis and gingivitis patients were more alkaline. An alkaline pH is associated with increased proteolytic activity of organisms that favour the deposition of calcium phosphate, which induces plaque mineralisation [22]. Furthermore, a higher oral pH can lead to the accumulation of dental plaque which, if not monitored by dental professionals, could deteriorate the dental enamel.

To date, only one study has assessed the leaching of manganese (Mn), Al, Ni, Cr, and Cu ions from fake Bs into AS at pHs of 4.9 and 7.8 and discovered that fake Bs release more Ni and Cr ions than standard orthodontic Bs [23]. This present study, however, found that the control and fake samples released almost similar concentrations of ions. Interestingly, the control samples released significantly more ions than their fake counterparts. More specifically, Na, Ca, and K ions concentrations of more than 100 mg/L in 28 days. Another study that immersed fake braces in other media; such as simulated bodily fluid; obtained a similar finding [11]. It is noteworthy that Ni ions were not detected in the AS, even in the AS of the control sample which is made of stainless steel, which contains Ni. Multiple factors that affect the quantity of metal ions released from a fixed orthodontic appliance; such as the galvanic corrosion of the metal, the corrosion resistance of the material, the effect of brazing or welding on the metals, and the surface texture of the samples; may explain why the control and fake samples released significantly different quantities of metal ions [23].

### Limitation of study

This present study was not without its limitations. Firstly, the AS was not replaced with an equal volume of AS. This may have caused the pH to be higher (> 7.4) than the pH of the AS at baseline pre-sample incubation. According to Mikulewics et al. [24], the constant flow of saliva in vivo dilutes any metal ions that have been released. As such, they developed a continuous flowing AS system that simulates the oral environment by maintaining a saliva flow rate of 0.5mL/min and a temperature of 37°C to test dental materials in vitro. Kovac et al. [25] and Karnam et al. [26] measured the cumulative concentrations of metal ions that orthodontic Bs and AWs release into AS over a 90-day period. Much like this present study, both these studies concluded that the quantity of metal ions leached did not surpass the recommended daily dosage.

Secondly, this present study only used one control sample. Therefore, future studies should use at least two to three control samples to increase the validity of the results. Thirdly, the fake samples mostly comprised passive fake braces, which customers are only supposed to use temporarily. Active fake braces, that bond to the teeth, would have yielded better results as they are placed in the oral cavity for longer periods of time. The results of this present study should be appraised with caution as the fake braces kits sold by non-dental online sellers did not include any content or composition information [16].

The fake and control braces released high concentrations of K, Ca, and Na ions, which indicates that these ions are a part of their composition. However, this present study did not examine if the percentage of ions released were proportional to the composition of the materials [16]. Nevertheless, based on the weights of the samples pre-incubation in AS and the designated doses of the AWs and Bs, the quantity of ions that each sample released could have been estimated if the actual composition of the sample was known, especially during the fabrication phase. As the fake braces did not contain any content or composition information, these calculations could not have been done accurately and the precise elemental composition of the fake braces could not have been determined. Although the samples did not release more than the recommended daily intake of each ion into the AS, precautions are still needed as minute changes in the quantity of ions released into the bloodstream can lead to several oral and bodily diseases. Therefore, it is crucial that future studies using saliva or blood from subjects with a history of using fake braces are aware and understand the possible diseases that may arise from the use of fake braces.

## Conclusions

The pH of the AS of the AW group differed significantly at Days 0, 1, 7, 14, 21, and 28. However, that of the B group only differed on Days 0 and 7. The finding of this present study indicate that fake braces release 10 different ions into saliva; namely, Na, K, Mg, Ca, Cu, Pb, Al, Li, Ba, and Cr. The concentrations of Na, Ca, and K ions released into the AS exceeded 100 mg/L during the immersion period. The concentrations of Ca and K ions released by the fake braces differed significantly from that of the control samples. However, the quantity of Li, Ba, and Cr ions that the control sample released did not differ significantly. The fake samples released significant more Na, K, Mg, Ca, Cu, Pb, and Al ions than the control samples. The findings of this present study sets the foundation for future studies on the potential risks and toxicity of wearing fake braces.

## Data Availability

The data underlying this article will be shared on reasonable request to the corresponding author.

## Abbreviations

ANOVA: Analysis of variance
SBF: Simulated body fluid
IOTN: Index of Orthodontic Treatment Need
